# Recent strategies of increasing metal tolerance and phytoremediation potential using genetic transformation of plants

**DOI:** 10.1007/s11816-017-0467-2

**Published:** 2018-01-03

**Authors:** Aleksandra Koźmińska, Alina Wiszniewska, Ewa Hanus-Fajerska, Ewa Muszyńska

**Affiliations:** 1 Institute of Plant Biology and Biotechnology, Faculty of Biotechnology and Horticulture, University of Agriculture in Krakow, Al. 29 Listopada 54, 31-425 Kraków, Poland; 2 Department of Botany, Faculty of Agriculture and Biology, Warsaw University of Life Sciences (SGGW), Nowoursynowska 159, Building 37, 02-776 Warsaw, Poland

**Keywords:** Environmental pollution, Genetic engineering, Genoremediation, Heavy metals, Hyperaccumulation, Transgenesis

## Abstract

Avoidance and reduction of soil contamination with heavy metals is one of the most serious global challenges. Nowadays, science offers us new opportunities of utilizing plants to extract toxic elements from the soil by means of phytoremediation. Plant abilities to uptake, translocate, and transform heavy metals, as well as to limit their toxicity, may be significantly enhanced via genetic engineering. This paper provides a comprehensive review of recent strategies aimed at the improvement of plant phytoremediation potential using plant transformation and employing current achievements in nuclear and cytoplasmic genome transformation. Strategies for obtaining plants suitable for effective soil clean-up and tolerant to excessive concentrations of heavy metals are critically assessed. Promising directions in genetic manipulations, such as gene silencing and *cis*- and intragenesis, are also discussed. Moreover, the ways of overcoming disadvantages of phytoremediation using genetic transformation approachare proposed. The knowledge gathered here could be useful for designing new research aimed at biotechnological improvement of phytoremediation efficiency.

## Introduction

Dynamic development of industrial, urban, and agricultural aspects of human activities in the 20th century resulted in chemical pollution of the environment (Gavrilescu et al. 2015). In many parts of the world, the concentrations of numerous metallic elements in soil and water have increased to a level that endangers human health (Kelishadi et al. 2014). Release of small metal particles into the air also constitutes an environmental hazard of great concern (Serbula et al. 2012; Kularatne and de Oliveira; Freitas 2013). To prevent and treat hazardous pollution, wide range of techniques for heavy metal removal has been developed. However, the conventional physicochemical remediation is expensive and detrimental to both microbiological life and soil properties (Fasenko and Edwards 2014; Singh and Santal 2015). Nowadays, apart from the traditional techniques involving mechanical or chemical treatment of a polluted substrate, science has supplied us with new opportunities of utilizing microorganisms (bioremediation), and plants (phytoremediation) to transform or remove toxic elements from the environment (Kang 2014; Mani and Kumar 2014; Gavrilescu et al. 2015). In numerous heavy metal-polluted locations, phytoremediation is a perspective technology of soil clean-up provided that plant genotypes with increased capability of stabilizing or extracting metals are employed. Phytoremediation techniques for treating metal-contaminated sites include phytoextraction, phytostabilization, rhizofiltration, and phytovolatilization (Ali et al. 2013; Lee 2013). Phytoextraction refers to an uptake of metals with further translocation and concentration in plant aboveground organs, whereas during phytostabilization, the toxic ions are maintained in the polluted substrate, being immobilizedin the roots. Heavy metals are thus stabilized in the rhizosphere. In addition, the vegetation cover prevents dust blow and water erosion of the ground (Pilon-Smits and Pilon 2002; Singh and Santal 2015). During rhizofiltration, contaminants are the root absorbed, concentrated, or precipitated from contaminated water or polluted effluents by the root system of phytoremediating plant (Raskin et al. 1997). Phytoextraction and rhizofiltration require harvesting of the plant biomass with accumulated toxic metals. The plant material is then usually combusted, and the produced ash may be used for metal recycling (Jabeen et al. 2009; Ali et al. 2013). An innovative trend in using the harvested biomass from metal hyperaccumulating plants is a preparation of “eco-catalysts” for chemical industry (Escande et al. 2014, 2015). Plants are also capable of removing inorganic pollutants through volatilization. In this process, metals are biologically converted to gaseous forms and released into the atmosphere (Ruiz and Daniell 2009; Kang 2014).

The main progress in screening for efficient phytoremediating plants was achieved by selecting genotypes (ecotypes) growing naturally in contaminated sites (Reeves and Brooks 1983; Pollard et al. 2014; Muszyńska et al. 2015). Some progress was also achieved after a long-term selection of heavy metal-tolerant clones in in vitro and greenhouse conditions (Iori et al. 2012; Wiszniewska et al. 2015; Muszyńska and Hanus-Fajerska 2017). Further improvement of responsive genotypes by genetic engineering may help in making phytoremediation feasible for heavily heavy metal-polluted sites. Modifications may involve transformation of plants with different genes responsible for metal transport and homeostasis, defense response to oxidative stress, or xenobiotic detoxification. The latter genes are especially widespread in bacteria, and are a target of the so-called genoremediation, i.e., engineering of bacterial genes to increase their remediation potential, with subsequent incorporation into plant genome (Mani and Kumar 2014). Achievements of the last decades suggest that genetic engineering can be valuable tool in improving phytoremediation potential of the plant material belonging to different taxonomic groups. Our intention was to provide a comprehensive review on the current achievements in both nuclear and cytoplasmic genome transformation towards enhanced plant tolerance to heavy metals and their suitability for soil clean-up. In this review, we have discussed the effects of overexpression of genes encoding metal transporters, metal chelators, and components of antioxidative system. The main novelty of our paper is presentation of potential transformation-based solutions to overcome the most important phytoremediation drawbacks, limiting its broad exploitation. For the first time, we provided the comprehensive review of cytoplasmic genome transformation for improved plant potential to effective soil clean-up. In addition, we have presented and discussed genes that would enable to produce plant material useful not only for phytoextraction, but also for phytostabilization of contaminated areas. We discussed perspective directions in genetic manipulations aimed at enhancing phytoremediation of heavy metals, such as gene silencing and *cis*- and intragenesis. The knowledge gathered here could be useful for designing novel research towards biotechnological improvement of phytoremediation efficiency.

## Advantages and shortcomings of phytoremediation techniques

There are numerous advantages of phytoremediation technology. The most important are low costs in comparison with the traditional remediation technologies, eco-friendly and landscape-friendly nature, or significant reduction of the volume of contaminated material for disposal (Lee 2013; Wan et al. 2016). As a typical in situ technique, it may be applied to a broad range of sites, even those previously neglected due to high cost of conventional remediation technologies. However, there are also several limitations of this green technology. According to Ali et al. (2013), the main drawback is a long time required for a clean-up, what is related to limited capabilities of plants to accumulate high levels of heavy metals and to sustain normal growth in toxic environment. Plant remediation potential is also limited due to slow growth and low biomass accretion. Therefore, phytoremediation can be effective in sites with relatively low pollution level and is restricted to places where contaminantsare reachable for the plant roots. In addition, phytoremediating plants are often unique ecotypes that inhabit specific ecosystems and their cultivation in other environmental conditions is difficult (Ali et al. 2013; Pollard et al. 2014). Finally, although public perception of phytoremediation is positive, improper biomass disposal may increase arisk of food chain contamination with heavy metals. Modification of plant genotypes via genetic engineering could help to overcome main shortcomings of phytoremediation and make it more efficient (Pilon-Smits and Pilon 2002). Therefore, we attempted to suggest some potential transformation-based solutions to the most important phytoremediation limitations. In Table 1, we present main challenges that should be confronted during soil clean-up using phytotechnologies, and propose solutions based on genetic transformation, supported by examples of recent studies in this field.

**Table 1:** Phytoremediation challenges and selected examples of transformation-based solutions

Phytoremediation challenges (acc. to Ali et al. 2013)	Examples of transformation-based solutions	Effects	References
Reduction of time required for clean-up	Accelerating heavy metal uptake by plants overexpressing genes encoding novel metal transporters Transporters of phytosiredophore–metal complexes: yellow stripe (YS1) and yellow stripe-like (YLS) Co-transformation with multiple gene constructs	In *N. tabacum*, BjYSL7 gene from *B. juncea* enhanced accumulation of Cd and Ni In *A. thaliana*, SnYSL3 from *S. nigrum* gene is up-regulated in the excess of Cd and enhances translocation of Cd to the shoots In *A. thaliana*, dual-gene transformation with phytochelatin synthase gene from garlic (AsPCS1) and YCF1 (yeast vacuolar transporter) gene increased Cd and As accumulation properties	Wang et al. (2013) Feng et al. (2017) Guo et al. (2012)
Increased bioavailability of the contaminants in the soil	Modulation of heavy metal uptake via transformation with components of bacterial efflux system Transformation with genes encoding components of *Pseudomonas putida* efflux system: CzcA, CzcB and CzcC Increasing heavy metal uptake from the soil due to secretion of phytosiderophores Potential candidate genes: nicotianiamine synthase (NAS) gene; nicotianamine amino transferase (NAAT) gene; deoxymugineic acid synthase (DMAS) gene	In *N. tabacum*, increased bioavailability of Cd and gene variant-dependent differences in Cd accumulation in plant organs In hypperaccumulator *A. hallerii* NAS regulates Zn bioavailability In *O. sativa*, HvNAS contributes to enhanced accumulation of Zn NAS and NAAT genes are expressed not only under Fe deficiency, but also in the presence of heavy metals (Cd, Pb and Ni)	Nesler et al. (2017) Tsednee et al. (2014) Masuda et al. (2008) Gupta and Singh (2017)
Accelerated growth rate and enhanced plant biomass accretion	Transformation-based organogenesis Transformation with rol genes from *Agrobacterium rhizogenes* Generation of hairy roots overexpressing modified copper resistance protein C (copC) gene from *Pseudomonas fluorescens* Stimulation of plant metabolism Transformation with heme oxigenase (HO) gene (regulation of phytochrome biosynthesis) Overexpression of isopentyl transferase (IPT) gene (increased synthesis of cytokinins)	In *S. americanus*, increased root biomass and plant tolerance to Pb and Cr In *N. tabacum*, increased root biomass, increased Cu accumulation and minimized toxicity symptoms In *B. napus*, higher plant biomass and increased tolerance of transformants to Hg stress, with decreased accumulation of Hg In *N. tabacum*, increased biomass accretion under Zn exposure	Alfaro-Saldaña et al. (2016) Perez-Palacios et al. (2017) Shen et al. (2011) Pavlikova et al. (2014)
Increased plant tolerance to different growth condition (i.e., elevated level of contaminants, climate, and light intensity)	Transformation towards enhanced general stress defense mechanisms Genes encoding components of antioxidant system Genes encoding transcription factors related to various abiotic stresses (salinity, drought, and cold) Genes encoding elements of DNA repair system Genes encoding cytochromes	In *A. thaliana*, overexpression of OsGSTL2 (glutathione S-transferase) gene confers tolerance to various heavy metals and other abiotic stresses Gene encoding DRAB (drought-responsive element binding) transcription factor increased Cu tolerance in transgenic *N. tabacum*; and Cd tolerance in transgenic *S. tuberosum* In *M. truncatula*, overexpression of MtTdp2α gene enhanced tolerance of plants exposed to Cu In *A. thaliana*, overexpression of CYP450-like gene (cytochrome P450) confers to tolerance to As, Cr, and Cd and other abiotic stresses	Kumar et al. (2013) Ban et al. (2011); Charfeddine et al. (2017) Faè et al. (2014) Rai et al. (2015)
Reduction of the risk of food chain contamination	The use of transgenic plants with increased ability to phytoremediation may itself accelerate soil clean-up and, an as a result, the risk of food chain contamination can be diminished		

## Strategies for improved efficiency of phytoremediation

To improve phytoremediation traits using transgenesis, the most popular strategy is to introduce and overexpressthe genes involved in the uptake, translocation, and sequestration of metals (Shukla et al. 2013; Mani and Kumar 2014; Das et al. 2016). Since metal metabolism in plant organism is extremely complex, there are numerous potential pathways that can be manipulated to increase metal accumulation in plant tissues, including metal mobilization and uptake by roots from the soil, formation of complexes with ligands and chelators, detoxification by deposition in vacuoles, and finally long-distance transport to shoots via symplast or apoplast (Nakamura et al. 2014; Das and Jayalekshmy 2015). The genes that are currently widely used to manipulate metal metabolism in plants are those that encode transporters of metal ions and metal-binding ligands (chelators).

### Overexpression of genes encoding metal transporters

Plants developed versatile systems responsible for metal uptake and transportation, to supply cells with essential microelements, such as Cu^2+^, Zn^2+^, Fe^2+^, and Co^2+^ (Williams et al. 2000). The plant genes encoding heavy metal transporters, usually represented by large gene families, are potential candidate genes for transformation towards improved phytoremediation potential. Manipulations involve enhancing of metal accumulation either in the roots for phytostabilization, or in the shoots for phytoextraction. Transformation may be also aimed at inhibiting heavy metal uptake and accumulation. Careful selection of suitable metal transporter genes is, therefore, linked with long-term purpose of the research, and some recent examples are briefly discussed in this section and presented in Table 2.

**Table 2:** Recent examples of transformation using genes encoding metal transporters for increased efficiency of heavy metal phytoextraction and phytostabilization

Metal transporter family	Gene product	Gene origin and host	Effects		References
Phytoextraction					
ATP-binding cassette (ABC)	AtATM3	*A. thaliana*/*B. juncea*	Increased accumulation of Cd and Pb in the shoots		Bhuiyan et al. (2011)
ABC	YCF1	*S. cerevisiae*/Populus alba x P. tremula var. glandulosa	Increased accumulation of Cd and Zn in the shoots		Shim et al. (2013)
Iron regulated/ferroportin (IREG/FNP)	PgIREG1	Psychotria gabriellae/*A. thaliana*	Increased accumulation of Ni in the shoots		Merlot et al. (2014)
Metal tolerance protein (MTP)	OsMTP1	*O. sativa*/*N. tabacum*	Increased accumulation of Cd in the shoots		Das et al. (2016)
Copper resistant protein (Cop)	copC	*Pseudomonas* sp./*A. thaliana*	Hyperaccumulation of Cu in the shoots		Rodriguez-Llorente et al. (2012)
Phytostabilization					
P-1b-ATPase, CDF	AtHMA4	*A. thaliana*/*N. tabacum*	Restricted Cd transport from the roots to the shoots		Siemianowski et al. (2014)
ZIP	AtZIP1, AtMTP1	*A. thaliana*/Manihot esculenta	Zn accumulation in the roots, with restricted transport to the shoots		Gaitan-Solis et al. (2015)
ABC	YCF1	*S. cerevisiae*/Populus alba x P. tremula var. glandulosa	Increased accumulation of Pb in the roots		Shim et al. (2013)

Tolerant transgenic plants may be obtained by manipulations concerning the efficiency of metal transport into a vacuole. There are several gene families that encode proteins functioning in cellular transport systems, and one of them is ATP-binding cassette (ABC) family, a large group of genes encoding proteins responsible for detoxification and ion-regulation processes (Martinoia et al. 2002). Protein transporters encoded by ABC genes were reported to be localized in the tonoplast of transgenic plant cells and to improve sequestration of metals in vacuolar lumen (Yazaki et al. 2006; Song et al. 2014). Advantageously, the effect of genetic transformation with ABC genes is directly linked with heavy metal mobility, and therefore, ABC genes could be overexpressed either to enhance translocation of mobile ions, like Cd and Cu, or to bind the non-mobile ones, like Pb, in the roots (Table 2). This way, plant ability to both phytoextraction and phytostabilization of heavy metals can be manipulated.

Genes belonging to cation diffusion facilitator (CDF) family encode various types of proteins called metal tolerance/transport protein (MTP), that enable cells to exclude excessive amounts of ions from cytoplasm. Plants overexpressing CDF genes produce elevated amounts of thiol compounds and, therefore, are able to efficiently sequester metal ions in the vacuoles by chelating them.This may result in hyperaccumulation of toxic ions in plant biomass. It was recently reported that during external Cd stress, overexpression of OsMTP1 gene from indica rice (*Oryza sativa* L. cv. IR64) in tobacco stimulated hyperaccumulation of cadmium and enhanced tolerance and accumulation of arsenic upon exogenous. As stress, indicating broad substrate specificity of OsMTP1 (Das et al. 2016). It is an important finding, since this feature may be exploited in genetic modifications of plants towards enhanced co-tolerance to various trace metals in multi-contaminated sites.

Metal ion transporters are also products of large gene family encoding ZRT/IRT-related proteins (ZIP), which are engaged in the regulation of cytoplasmic transport of zinc and iron. In wild-type plants, these high affinity uptake systems are induced at transcriptional level by Zn or Fe deficiency (Eng et al. 1998). Majority of ZIP proteins form transmembrane ion channels with histydyl-rich metal-binding sites (Eng et al. 1998). Numerous recent studies focused on the exploitation of ZIP genes in crop biofortification with Zn and Fe (Tiong et al. 2015). However, these genes may be also overexpressed to improve phytoremediation of soil containing excessive amounts of heavy metals.In a model study, Conolly et al. (2002) revealed that overexpression of a geneencoding AtIRT1 in *A. thaliana* caused about 150% increase in accumulated amount of cadmium and zinc. More recent approach includes the use of metal transporters obtained from hyperaccumulator species, like *Noccaea cearulescens*, which contributes to higher efficiency of soil clean-up. For instance, overexpression ofNcZNT1 transgene encoding Zn transporter resulted in an enhanced accumulation of Zn and Cd in the transformed plants of *A. thaliana* (Lin et al. 2016). However, some ZIP proteins seem to be highly specific, and overexpression of their genes does not always result in improved accumulation of toxic heavy metals, even close analogues, like cadmium (Tiong et al. 2014). Therefore, another strategy exploited transformation with other genes encoding metal transporters and engaged in the uptake and transport of microelements. These genes may downregulate or upregulate the expression of ZIP genes, thus modifying the ability of transformed plants to accumulate toxic heavy metals. Usually, heavy metal uptake is significantly reduced due to increased concentration of microelements. This phenomenon can be useful in both phytoremediation of metal-contaminated soil, and crop biofortification in essential metals (Gong et al. 2015; Siemianowski et al. 2014).

### Overexpression of genes encoding metal chelators

The main classes of metal chelators include phytochelatins (PCs), which are peptides enzymatically synthesized from glutathione, low molecular organic acids and amino acids (LMWOA), and metallothioneins (MT). Among these compounds, only metallothioneins are direct products of gene expression. In contrast, the level of phytochelatins and organic and amino acids depends on the activity of enzymes involved in their biosynthesis, and thus genes encoding these enzymes should be manipulated.

Two key enzymes in the synthesis of PCs are phytochelatin synthase (PCS) and *c*-glutamyl cysteine synthetase (*c*-GCS) (Hirata et al. 2005). There are numerous examples of enhanced tolerance to heavy metals in transformed plants overexpressing phytochelatin synthase gene. Depending on the origin of the transgene, model plants of tobacco (*Nicotiana glauca* and *Nicotiana tabacum*) were either capable of greater accumulation of Cd and Pb, or were simply more tolerant to Cd, although without enhanced accumulation of this element (Huang et al. 2012; Chen et al. 2015). A serious concern of this approach is that various PCS differentially affect heavy metal transport within transformed plants. For example, PCS gene from *Populus tomentosa* reduced cadmium translocation to the aerial parts of tobacco (Chen et al. 2015), while PCS from aquatic macrophyte Ceratophyllum demersum enhanced this process (Shukla et al. 2013). An efficient way to overcome this issue could be co-transformation directed at multiple overexpression of genes involved in metal homeostasis. This approach may also increase the possibility to produce efficient hyperaccumulator. Guo et al. (2012) transformed *A. thaliana* with two genes: PCS, responsible for phytochelatin synthesis, and YCF1, ABC metal transporter, and obtained plants exhibiting co-tolerance to Cd and As, with increased bioaccumulation of these trace metals in vacuoles. Overexpression of ABC transporter gene reduced hypersensitivity reaction related to the accumulation of thiol-metal chelates, which, in turn, ameliorated plant tolerance. In addition, Zhao et al. (2014) proved that co-transformation with PCS and glutamyl cysteine synthetase (GCS) genes was beneficial to a transformation with PCS gene alone. They reported that overexpression of GCS enhanced the activity of PCS, resulting in higher production of phytochelatins. Due to this stimulatory effect, double transformants over-performed both wild-type plants and single transformants in relation to Cd accumulation and translocation.

Metal chelating properties were also reported for numerous organic low-molecular-weight compounds, such as organic acids and non-proteinogenic amino acids. Increased synthesis of these substances is often associated with plant tolerance to an excess of heavy metals (Lopez-Bucio et al. 2000). The genes encoding enzymes active in organic acid and amino acid synthesis can be also manipulated to improve phytoremediation process. A classical example could be an incorporation of HvNAS1 gene from *Hordeum vulgare* into *Arabidopsis*, reported by Kim et al. (2005). The gene encodes nicotianamine synthase, an enzyme that catalyzes trimerization of *S*-adenosyl methionine to nicotianamine, an amino acid capable of metal chelating. Due to increased biosynthesis of nicotianamine, the transformants accumulated Ni more efficiently than wild-type plants and were capable of growing on Ni-rich serpentine soil without symptoms of Ni toxicity. This pioneer study revealed the role of nicotianamine in the mechanism of nickel detoxification by the formation of Ni–NA complexes that may be either deposited in vacuoles or translocated to the upper parts via phloem and xylem. Metals could be also chelated by organic acids, such as citric acid and oxalic acid. Although exudation of these compounds from the roots is specifically induced by aluminum, other stresses, including heavy metal exposure, also stimulate this process (Ding et al. 2014). In recent years, numerous genes related to organic acids exudation were identified, and, consequently, transformation experiments towards improved Al tolerance in plants were conducted (Wang et al. 2010, 2012; Zhou et al. 2014). Increased production of organic acids may be achieved by overexpression of the gene encoding enzymes catalyzing their metabolism, such as malate dehydrogenese, citrate synthase (CS), and phosphoenolpyruvate carboxylase (PEPC) (Wang et al. 2010, 2012). Another way may be the overexpression of genes encoding citrate transporters, such as those belonging to multidrug and toxic compound extrusion (MATE) family. In transformed plants, Al ions are effectively chelated in the root system, where citrate is released, and, therefore, toxic ions remain stabilized in the rhizosphere (Zhou et al. 2014). It was recently shown that overexpression of aluminum-induced protein (AIP) gene enhanced a tolerance of transformed *A. thaliana* plants to various heavy metals, including Cd and Cu (Jang et al. 2014). Based on these findings, a new strategy in phytoremediation area may be an exploitation of genes related to Al tolerance aimed at the enhancement of heavy metal chelation and stabilization.

Metallothioneins (MTs) are ubiquitous cysteine-rich proteins capable of high affinity coordination of heavy metal ions via cysteine residues gathered in Cys–X–Cys or Cys–Cys motifs. Plant metallothioneins are currently categorized into four types that slightly differ in a structure of cysteine-rich domains (Leszczyszyn et al. 2013). The role of plant MTs is generally attributed to the homeostasis of essential metals (Se, Zn, Ni, and Cu), and detoxification of xenobiotic metals (Cd, Hg, and Ag) (Tripathi et al. 2015). In recent years, great number of MT and MT-like gene sequences became available in databases and numerous studies reported on improved heavy metal tolerance in plants overexpressing MTs transgenes (Leszczyszyn et al. 2013). These reports were comprehensively reviewed and excellently discussed previously, for example by Eapen and Souza (2005), Kotrba et al. (2009), and Yadav et al. (2010). Therefore, here, we have just overviewed some very recent examples and presented them in Table 3. Instead, an interesting approach worth discussing is plant transformation with MTs genes isolated from halophytes. This group of plants includes species adapted to high salinity. Recent studies revealed that the mechanisms enabling plant growth under salt stress ameliorate also plant response to other abiotic stresses, including heavy metal treatment (Mariem et al. 2014; Taamalli et al. 2014; Guo et al. 2017). Metalothionein gene IlMt2a was successfully identified and characterized in a halophyte *Iris lactea* var. *chinensis* (Gu et al. 2014). Its subsequent incorporation into *Arabidopsis* genome resulted in higher tolerance of transgenic plants exposed to cadmium and copper (Gu et al. 2014, 2015). Heavy metal tolerance seemed to be associated with reduced production of reactive oxygen species (ROS), indicating high effectiveness of antioxidant defense system. Similar mechanisms led to increased heavy metal tolerance of tobacco transformed with SbMt2 gene from *Salicornia brachiata*, an extreme halophyte (Chaturvedi et al. 2014). Transgenic plants were capable of maintaining cellular homeostasis by modulating ROS scavenging/detoxification. Moreover, overexpression of SbMt2 gene was found to enhance Zn translocation into shoots, and therefore, it could be a good candidate gene for boosting stress tolerance and phytoremediation effectiveness via transgenesis (Chaturvedi et al. 2014). Future studies on genetic transformation towards improved phytoremediation potential could be significantly accelerated thanks to elucidating the mechanisms that allow extremophytes to survive in highly stressful conditions.

**Table 3:** Recent examples of transformation using genes encoding metallothioneins (MTs) for improved heavy metal tolerance and accumulation in plants

Gene	Origin	Host	Effect	References
EhMT1	*Elsholtzia haichowensis*	*Nicotiana tabacum*	Higher tolerance to Cu and higher accumulation Cu in roots	Xia et al. (2012)
$\alpha$MT1a	*Linum usitatissimum* L.	*Linum usitatissimum* L.	Overexpression of Cd-binding peptids enhanced accumulation of cadmium	Vrbova et al. (2015)
SpMTl	*Sedum plumbizincicola*	*Sedum plumbizincicola*	Elevated SpMTL transcript level might contribute to the trait of Cd hyperaccumulation and hypertolerance	Peng et al. (2017)
rgMT (type I)	*Oryza sativa* L.	*Sacharomyces cerevisiae* *A. thaliana*	Improved seed germination rates and increased biomass in the presence of heavy metal salts	Jin and Daniell (2014)
BcMT1 BcMT2	*Brassica campestris*	*Arabidopsis thaliana*	Enhanced tolerance to Cd and Cu and increased Cu concentration in the shoots; decreased Production of Cd- and Cu-induced ROS, thereby protecting plants from oxidative damage	Lu et al. (2015)
OsMT2c	*Oryza sativa* L.	*Arabidopsis thaliana*	Improved tolerance to Cu stress and increased ROS scavenging ability	Liu et al. (2015)
SaMT2	*Sedum alfredii*	*Saccharomyces cerevisiae*/*Nicotiana tabacum*	Increased Cd tolerance and accumulation	Zhang et al. (2014)
ThMT3	*Tamarix hispida*	*Salix matsudana*	Increased tolerance to Cu stress and higher NO productions (what contributed to the induction of adventitious roots under Cu stress)	Yang et al. (2015)
ThMT3	*Tamarix hispida*	*Saccharomyces cerevisiae*	Enhanced Cd, Zn, and Cu tolerance	Yang et al. (2011)

## Strategies for improved heavy metal tolerance

Genetic transformation allows for obtaining plants exhibiting heavy metal resistance by manipulation with metabolic pathways responsible for stress defense reactions. At the cellular level, tolerance is usually manifested by an induction of mechanisms involved in DNA repair and radical scavenging. Using genetic manipulation of these two traits, one may further stimulate plant tolerance to toxic elements. The most common strategy in the studies on heavy metal stress is enhancement of plant antioxidant activity.

### Overexpression of genes encoding components of antioxidant machinery

The oxidative damage is a consequence of various abiotic stresses, and occurs through the accumulation of elevated level of reactive oxygen species (ROS). Damaging effects of ROS to biomolecules, such as DNA, proteins, and lipids, can be minimized by antioxidant defense system including enzymatic mechanisms based mainly on superoxide dismutase (SOD), ascorbate peroxidase (APX), catalase (CAT), and glutathione *S*-transferase (GST) (Dixit et al. 2011; Hellou et al. 2012). It is, therefore, a popular approach in genetic transformation towards improved phytoremediation properties to increase the activity of antioxidant enzymes and ROS scavengers. Transformation experiments directed at increasing general tolerance to abiotic stresses via overexpression of genes encoding antioxidant enzymes were conducted with various transgenes and host plants. However, the results related to the response to heavy metals were rather confusing. It was found that during an exposure to Cu, Cd, and As, transgenic plants overexpressing SOD, APX, and CAT genes generated lower amounts of ROS than wild-type plants, and the activity of antioxidant enzymes was highly elevated under stress (Lee et al. 2007; Guan et al. 2009; Gao et al. 2016). Nevertheless, the transgenic plants often exhibited both morphological and physiological disturbances such as growth retardation, abnormal root architecture, and reduction of photosynthetic efficiency at normal temperature condition (Iannone et al. 2015; Gao et al. 2016). Iannone et al. (2015) suggested that catalase did not play a crucial role in protecting tobacco plants against Cd toxicity. Instead, transgenic plants engineered for decreased activity of constitutive CAT (line CAT1AS) exposed to Cd were capable of activating alternative defense mechanisms against Cd stress, based mainly on increasing the activity of constitutive guaiacol peroxidase and ascorbate peroxidase. Moreover, Gao et al. (2016) reported that transformed plants of *Sedum alfredii* had reduced ability to accumulate cadmium ions than wild-type plants.

Contradictory results were also obtained for overexpression of glutathione *S*-transferase (GST) genes in plants subjected to heavy metal stress. These enzymes, possessing glutathione peroxidase activity, catalyze conjugation reactions between glutathione and a number of xenobiotics, and thereby contribute to the detoxification of free radicals (Hellou et al. 2012). Although transgenic tobacco plants expressing Trichoderma virens GST gene became more tolerant to cadmium ions and showed reduced lipid peroxidation in comparison with wild-type plants, Cd accumulation was not improved (Dixit et al. 2011). Growing transgenic alfalfa plants co-expressing GST and human cytochrome P450 2E1 genes turned out to be more efficient. The plants were more resistant to the toxic effects of heavy metals (Cd and Hg) and organic xenobiotic trichloroethylene and accumulatedgreater amounts of these pollutants than non-transgenic control plants (Zhang and Liu 2011; Zang et al. 2013). Such a combination of genes may be potentially used for phytoremediation of soils contaminated with a mixture of heavy metals and organic pollutants.

Attempts were also made to enhance synthesis of polyamines, since these compounds play an important role in the reduction of oxidative cell damage (Wen et al. 2010). For this purpose, a transgenic European pear (*Pyrus communis* L. ‘Ballad’) line was produced overexpressing apple spermidine synthase MdSPDS1 gene. After exposure to Cd and Zn, the transformants responded with enhanced synthesis and accumulation of polyamines as compared with wild-type plants. Beneficial effects were attributed to antioxidant activity of spermidine and its ability to bind free metal ions (Wen et al. 2010). The essential role of polyamines in the reduction of stress symptoms in *Pyrus communis* was further confirmed during salt and cadmium treatment of transgenic lines containing MdSPDS1 in antisense orientation (Wen et al. 2011).

The main issue with enhancing heavy metal tolerance through overexpression of genes encoding antioxidant system is that there is not one defense mechanism against oxidative stress and the system of antioxidant response to the same heavy metal might differ in various plants and even in various organs and tissues within a plant organism. Schneider et al. (2013) provided an evidence for a cell type-specific antioxidant activity under Zn treatment and proved that epidermal cells exhibited higher accumulation of both glutathione and S-transferase proteins than the mesophyll tissue of *Noccaea caerulescens* leaves. Therefore, the impact of tissue-specific gene expression should be taken into consideration during genetic modification of plants for biotechnological purposes. Besides, antioxidant enzymes may be identified as different isoforms of the same proteins, some of which may be up-regulated, while other down-regulated in response to heavy metals (Alvarez et al. 2009). Moreover, regulation of genes engaged in antioxidant reactions additionally depends on stress exposure, and thus, various antioxidant molecules are activated with the lapse of time (Ovečka and Takáč 2014).

The latest reports point at the possibility of stimulating plant heavy metal tolerance by overexpression of genes related to DNA repair and transcription. In this case, the mechanisms of tolerance are related to up-regulation of other genes, mainly those encoding antioxidant enzymes, such as cytosolic and chloroplastic isoforms of SOD and CAT, metallothioneins and metal transporters, as well as enzymes of DNA repair system (Faè et al. 2014; Charfeddine et al. 2017). Based on this, increased tolerance to Cu was obtained in transgenic *Medicago truncatula* overexpressing DNA repair gene MtTdp2α, encoding tyrosyl-DNA phosphodiesterase 2 (Faè et al. 2014), and to Cu and Cd in potato overexpressing genes encoding transcription factors from drought-responsive element binding (DREB) family (Ban et al. 2011; Charfeddine et al. 2017). These examples showed that stress tolerance in plants is a very complex system and that efficient engineering of plants towards improved phytoremediation requires mechanisms involved in the cross-talk between heavy metals and plant response that remain to be explored in the future studies.

## Transformation of chloroplast genome towards improved phytoremediation

Small chloroplast genome can be modified by genetic transformation, generating cells and plants with transgenic plastid genomes, which are referred to as transplastomic plants. A specific feature of plastid transformation is homologous recombination that facilitates the site-specific alteration of endogenous plastid genes as well as the precisely targeted insertion of foreign genes into the plastid DNA (Bock 2015). This enables also overcoming common disadvantages of DNA transformation, such as gene silencingand position effect (Daniell et al. 2016). Homologous recombination has been used to analyze chloroplast gene functions and expression at all levels in vivo, and now, this method is nearly comparable to the techniques used for plant nuclear transformation (Lu et al. 2013; Bock 2015; Daniell et al. 2016). Benefits of chloroplast transformation include also the fact that codon optimization is not required to improve expression of bacterial transgenes, and therefore, any bacterial gene can be inserted in chloroplast genome (Quesada et al. 2005). An additional advantage of plastid transformation is minimizing the risk of transient gene transfer in the environments, since chloroplast DNA is maternally inherited and the dissemination of plastid DNA via pollen is rare phenomenon in plants (Maliga 2002). Chloroplast transformation is especially suitable for the development of transgenic plants when multiple genes are required for effective phytoremediation, and therefore, it is an ideal approach for mercury phytoremediation. Pioneer studies revealed that transformation of tobacco chloroplast genome by native bacterial genes merA and merB induced plant resistance to very high concentrations, up to 400 mM, of phenylmercuric acetate (PMA). Transgenic plants not only survived such high concentrations of PMA but also grew better than control untreated plants (Ruiz et al. 2003; Hussein et al. 2007). Although it was reported that organic Hg is transported in plants more efficiently than inorganic forms of mercury (Kumar et al. 2004), transgenic tobacco volatilized Hg^0^ efficiently, independently of the form of Hg in the soil, confirming that both mercuric ion reductase and organomercuriallyase were active in transgenic chloroplasts (Hussein et al. 2007). Nevertheless, a disadvantage of the described strategy is that elemental mercury is volatilized from the cell and released back into the environment, where it can be converted into highly toxic forms. For this reason, attempts had been made to stimulate mercury accumulation in plant tissues for their subsequent utilization (Ruiz et al. 2011; Zhang et al. 2013). Ruiz et al. (2011) reported on a development of a transplastomic system to express murine metallothionein gene (MT1) that enabled accumulation of mercury in high concentrations within tobacco cells. The transgenic lines were resistant up to 20 μM mercury, maintained high chlorophyll content and biomass, and actively translocated Hg to the leaves, indicating increased phytoremediation capacity. Chloroplast transformation may also increase plant tolerance to stress induced by excessive concentration of copper. Jin and Daniell (2014) reported that overexpression of γ-tocopherolmethyltransferase gene (γ-TMT) in transformed tobacco chloroplasts resulted in accumulation of the enzyme in the inner envelope membrane. Due to increased activity of the enzyme, a concentration of α-tocopherol in transplastomic seeds was twice as high as in wild-type ones. As a consequence, transplastomic plants grew better in the presence of excessive amounts of copper, exhibiting increased tolerance related to antioxidant defense (Jin and Daniell 2014). Martret et al. (2011) also attempted to stimulate plant antioxidant system under cadmium and zinc treatment by transformation of tobacco chloroplast genome with three genes encoding antioxidant enzymes: dehydroascorbatereductase (DHAR), glutathione *S*-transferase (GST), and glutathione reductase (GR). Although transplastomic plants were less sensitive to various stress conditions, such as increased salinity and chilling, chloroplast transformation did not ameliorate plants response to heavy metal exposure. This illustrates the fact that in some cases, nuclear transformation has more pronounced impact on antioxidant system than chloroplast transformation. Therefore, screening for appropriate genes is essential to successfully transform plastids towards heavy metal tolerance. A good candidate gene is BrMT1 encoding *Brassica rapa* type-1 metallothionein. This gene was successfully targeted to the chloroplasts of *Arabidopsis* and its overexpression in either the chloroplasts or the cytosol allowed for effective detoxification of cadmium in transplastomic plants (Kim et al. 2007). In particular, the chloroplast-targeted BrMT1 was associated with a significant reduction in chlorosis and accumulation of H_2_O_2_. Considering efficiency of plastid transformation, there are some concerns about the functioning of transgenic chloroplasts in the roots, where are naturally absent. Since root system plays a crucial role in phytoremediation, Davison (2005) doubts whether chloroplast transformation would be effective tool for enhanced heavy metal uptake by this organ. This problem could be by-passed by elaboration of transformation protocols for other types of plastids, especially leucoplasts, that are present also in organs that are not active in photosynthesis. Recently, similar approach has been successfully applied in creating transplastomic *Nicotiana* plants exhibiting elevated tolerance to multiple biotic and abiotic stresses (Chen et al. 2014).

## Miscellaneous directions of transformation towards improved phytoremediation

Algal species, especially microalga, have great ability to absorb and transform pollutants, including heavy metals. These organisms synthesize both phytochelatins and metallothioneins, and, therefore, are capable of chelating and efficiently detoxifying toxic elements (Perales-Vela et al. 2006). However, the algae have not been widely studied and exploited for soil remediation purposes, since they naturally dwell in aquatic environments. Nevertheless, due to their unicellular structure, algal cells may serve as a tool in fundamental research studying cellular mechanisms of heavy metal tolerance. They can also be a source of novel genes to be used in plant transformation. A good example is *Chlamydomonas*, in the genome of which at least 11 unique gene families known to encode metal transporters were described (Rubinelli et al. 2002). Another interesting feature is specific cell wall structure in alga, which contains sulphated oligosaccharides playing a role in heavy metal binding (Xue et al. 1988). Investigation in this field opens new possibilities of engineering plant cell wall proteins to increase their metal-binding capacity.

An emerging transformation approach for increased heavy metal resistance may become gene silencing. Biological basis for this process is RNA interference, a conserved defense mechanism, in which small RNA molecules suppress gene expression by interfering with the translation of target mRNA (Saurabh et al. 2014). This technique was applied to improve crop quality in several important traits, such as allergen elimination, altered plant phenotype, biofortification, seedless fruit production, and more (see review of Saurabh et al. 2014). Gene silencing allows for avoiding heavy metal accumulation, as reported by Li et al. (2007), where Cd content in grains was drastically reduced by silencing phytochelatin synthase gene. However, gene silencing via RNA interference may also facilitate heavy metal uptake, resulting in hyperaccumulation of toxic elements. Dhanker et al. (2006) reported that *Arabidopsis* plants silenced for arsenate reductase gene were capable of efficiently translocating arsenic to the shoots, in contrast with wild-type plants (Dhankher et al. 2006). Enhanced Cd translocation to the shoots was also achieved in rice by silencing the gene encoding root-localized Cd-transporter OsNRAMP5 (Takahasi et al. 2014). Such an approach would help to intensify phytoextraction of metallic pollutants from the soil. As gene silencing technology has numerous benefits, such as precision, efficiency, stability, and flexibility (Saurabh et al. 2014), substantial increase in phytoremediation efficiency can be expected in the nearest future.

## Future prospects to overcome transformation concerns

Genetic engineering is a powerful tool that may accelerate development of new plant lines with traits desirable in phytoremediation. However, there have been public concerns about testing transgenic lines in field conditions, and therefore, no genetically modified plants have been tested in large-scale field experiments for their specific potential in heavy metal remediation. Thus, also the potential risks associated with outcrossing with any wild relatives or uncontrolled spread due to higher tolerance level have never been clearly evaluated. The risk of gene escape with engineered hyperaccumulator lines can be reduced if plant material is applied in isolated industrial districts instead of agricultural areas. A novel solution can be an application of cisgenesis and intragenesis in genetic engineering of phytoremediating plants. These new tools in plant genetic modification were successfully applied in improving crop quality and enhancing tolerance to biotic stresses (Espinoza et al. 2013). In both methods, plants are modified with the copies of natural genes: in cisgenesis, the construct must contain complete genetic elements from a sexually compatible gene donor (promoter, coding sequence including introns, and terminator), while in intragenesis, the constructs may contain genetic elements from different genes within a sexually compatible gene pool (Espinoza et al. 2013). Cisgenic and intragenic approach may be especially valuable in breeding programs that would facilitate biomass production and growth of natural hyperaccumulators. Plants belonging to Brassicaceae family, such as *Arabidopsis* or *Thlaspi*, are good candidates for genetic modifications due to scientific knowledge on their genetics and their close phylogenetic relationships. Genetic foundations of heavy metal tolerance were extensively studied in this family and several ecotypes of hyperaccumulator species were identified (Marmiroli et al. 2006). Recent findings in genetic mapping in maize may facilitate a production of cisgenic plants with enhanced/limited ability to accumulate cadmium in leaf tissues, which can be exploited in phytoremediation of contaminated soil (Zdunić et al. 2014). Although, to date, there have been no reports exploiting cisgenesis and/or intragenesis in modulating plant responses to heavy metal stress, these novel techniques provide an excellent opportunity to overcome concerns with transgenesis and accelerate studies on genetic manipulations towards increased efficiency of phytoremediation.

## Conclusions

In this review, we have comprehensively discussed recent achievements and novel directions of plant transformation towards enhanced heavy metal tolerance and phytoremediation. We also proposedsome potential transformation-based solutions to the most important phytoremediation drawbacks. In this view, plant transformation may facilitate soil clean-up for sustainable environmental protection. Since genetic engineering may be extremely powerful tool in environmental studies, especially taking into account that new technologies, such as genome editing and genome engineering, are still emerging, the knowledge gathered here could be useful in designing new research towards biotechnological improvement of phytoremediation efficiency.
